# Abstract of the Proceedings of the Medical Society of South Western New York, Held at Jamestown, May 10, 1865

**Published:** 1865-05

**Authors:** J. Andrews

**Affiliations:** Secretary; Jamestown


ART. III.—*Abstract of the Proceedings of the Medical Society of South Western New York, held at Jamestown, May* 10, 1865.

*Dr. Irwin* of Dunkirk, reported a case of Cystic Sarcoma of Liston, the tumor involving one testicle and cord, and weighing 2 ℔s and 10 ounces. It was removed about the first of November last, and the patient made a good and rapid recovery, and in four or five weeks was at work as a day laborer. Dr. Irwin then hoped it would not return, but late in the winter it made its appearance in the inguinal canal, and is rapidly doing its fatal work.

*Dr. Hazeltine* of Jamestown, reported the spontaneous expulsion of a large uterine tumor. A young married lady was confined with twins; nearly two years afterwards she had an abortion with twins; some three or four months afterwards she was taken sick as if in labor, and after long continued and severe pains, expelled this tumor, and since has been well. She had no hemorrhage; the tumor was organized, with a shining, firm covering, pyriform in shape, and five lines in diameter at neck, where it separated.

*Dr. Strong* of Westfield, made a report upon epidemic erysipelas, as it occurred in the town of Ripley last March. (See Art. iv. of this Journal.) He thought it an asthenic disease, depending upon a blood poison, and asked discussion upon the report.

*Dr. Loop* of North East, Pa., the adjoining town, had seen a few cases in his practice; viewed the disease as asthenic, and the treatment was about the same.

*Dr. Cochrane* of Westfield, had seen a few cases of it also, and agreed with Dr. Strong as to pathology and treatment. The first case he called diphtheria at his first visit, and looked for a membraneous deposit the next visit, but instead of that found an erysipelas of the head making its appearance.

*Dr. Bennett* of Erie, Pa., had seen several of the worst cases reported by Dr. Strong, and coincided fully in the report.

*Dr. Hazeltine* referred to a similar epidemic, occurring some twenty years since in the same town, but extending more generally through the county. Said that at that time it took a more sthenic type, and the patients bore depletion, even venesection, afterwards tonics and stimulants. That epidemic took the name of "black tongue," and was very fatal.

*Dr. Bennett* spoke of a Thompsonian Doctor, who came to Ripley, whilst that epidemic was proving most fatal, and acquired a permanent reputation by his success with "Capsicum, No. 6," etc.

*Dr. Smith* of Dunkirk, narrated his early experience, having commenced his practice in such an epidemic. He was driven by the fatality of the disease in the hands of physicians who were depleting, to adopt another course, and went to using stimulants and general sustaining treatment. He then learned not to deplete in *epidemic erysipelas*.

*Resolutions on the occasion of the death of Dr. C. E. Washburn of Fredonia, N. Y.*

A Committee, consisting of Drs. Strong, Bennett and Irwin, was appointed to present to the Society resolutions in reference to the death of Dr. C. E. Washburn, of Fredonia. They reported the following, which were unanimously adopted:

*Resolved*, That the Secretary of this Society be instructed to enter upon the records the following notice of the death of Dr. C. E. Washburn:

Died, in the service of his country, CHARLES E. WASHBURN, M.D. an honored founder and member of the Medical Society of South Western New York.

Dr. Washburn was a man of rare and scholarly attainments in medical science and general literature; a graduate, and subsequently a Tutor in Amherst College, Massachusetts; a pupil of Dr. Valentine Mott; an Assistant-Surgeon in the New York City Hospital for two years; an enthusiast in medical research, he entered upon his profession with an amount of experience and culture vouchsafed only to the favored few. Voluntarily offering himself to his country, he fell a victim to exposure and untiring labor in camp and field, ministering to our prisoners dying from the pestilence of malignant fever on their way from the charnel-house of Andersonville.

*Resolved*, That as medical men, members of this Society, we have lost one of our most worthy and loved associates; we mourn his death, and will cherish his memory as a noble and cultivated physician, a pure patriot, a living Christian.

*Resolved*, That we tender to his wife and children our heartfelt sympathy, and would share with them their grief in this their great bereavement.

*Resolved*, That a copy of these resolutions be sent to the family of our deceased brother, and to the Buffalo Medical Journal for publication.

J. ANDREWS, Secretary.

Jamestown, May 17, 1865.

